# HIV viral suppression in the era of dolutegravir use: Findings from a national survey in Tanzania

**DOI:** 10.1371/journal.pone.0307003

**Published:** 2024-08-14

**Authors:** Doreen Kamori, Godfrey Barabona, Werner Maokola, Joan Rugemalila, Macdonald Mahiti, Mucho Mizinduko, Amon Sabasaba, George Ruhago, Linda Mlunde, Salim S. Masoud, Davis Amani, Erick Mboya, Sabina Mugusi, Anath Rwebembera, George Mgomella, Sarah Asiimwe, Beatrice Mutayoba, Prosper Njau, Takamasa Ueno, Andrea Pembe, Bruno Sunguya

**Affiliations:** 1 Department of Microbiology and Immunology, Muhimbili University of Health and Allied Sciences, Dar es Salaam, Tanzania; 2 Collaboration Unit for Infection, Joint Research Center for Human Retrovirus Infection, Kumamoto University, Kumamoto, Japan; 3 Division of Infection and Immunity, Joint Research Center for Human Retrovirus Infection, Kumamoto University, Kumamoto, Japan; 4 National AIDS Control Programme, Dodoma, Tanzania; 5 Department of Internal Medicine, Muhimbili National Hospital, Dar es Salaam, Tanzania; 6 Department of Epidemiology and Biostatistics, Muhimbili University of Health and Allied Sciences, Dar es Salaam, Tanzania; 7 Department of Development Studies, Muhimbili University of Health and Allied Sciences, Dar es Salaam, Tanzania; 8 Department of Community Health, Muhimbili University of Health and Allied Sciences, Dar es Salaam, Tanzania; 9 Department of Clinical Pharmacology, Muhimbili University of Health and Allied Sciences, Dar es Salaam, Tanzania; 10 US Centers for Disease Control and Prevention, Dar es Salaam, Tanzania; 11 The Global Fund, Tanzania; 12 Department of Obstetrics and Gynecology, Muhimbili University of Health and Allied Sciences, Dar es Salaam, Tanzania; Nigerian Institute of Medical Research, NIGERIA

## Abstract

**Background:**

Tanzania has made significant progress in improving access to HIV care and treatment. However, virologic suppression among people living with HIV (PLHIV) has not been fully realized. In March 2019, Tanzania introduced a World Health Organization (WHO)—recommended dolutegravir-based regimen as the default first-line regimen. Eighteen months later we investigated the HIV viral suppression rates and the factors associated with lack of viral suppression among PLHIV (children and adults) in Tanzania.

**Methodology:**

A cross-sectional survey was conducted from September to December 2020 among PLHIV on antiretroviral therapy (ART) in Tanzania. Whole blood samples, demographic data and clinical information were obtained from eligible adults (≥15 years) and children (< 15 years) attending thirty-six HIV care and treatment centres located in 22 regions of Tanzania mainland. A whole blood sample from each participant was processed into plasma and HIV viral load was estimated using real-time PCR. HIV viral suppression was defined at a cut-off of < 50 copies/mL as recommended by WHO. Analyses were conducted using descriptive statistics to establish the national representative prevalence of viral suppression, and logistic regression analyses to determine independent factors associated with non-suppression.

**Results:**

A total of 2,039 PLHIV on ART were recruited; of these, adults and children were 57.5% (n = 1173) and 42.5% (n = 866), respectively. Among the adult population, the mean age and standard deviation (SD) was 42.1 ± 12.4 years, with 64.7% being female. Among children, the mean age and SD were 9.6 ± 3 years, and 53.2% were female. Overall viral suppression at < 50 copies/mL (undetectable) was achieved in 87.8% of adults and 74.4% of children. Adults and children on dolutegravir-based regimen recorded viral suppression rates of 89.7% and 85.1% respectively. Factors independently associated with lack of viral suppression status in the adult population were age and ART adherence while in the children population, the factors were sex, ART adherence, and current ART regimen (p<0.05).

**Conclusion:**

Dolutegravir-based regimens are promising to help attain epidemic control in Tanzania. More efforts especially on ART adherence are needed to attain optimal treatment outcomes for children and adults PLHIV in Tanzania.

## Introduction

Tanzania is among the countries with a high burden of Human Immunodeficiency virus (HIV) infection [1]. Various strategic efforts have been implemented to address this burden leading to a steady decline of new HIV infections, morbidity, and related mortality in the country [1]. Despite such success, optimum treatment outcome in People Living with HIV (PLHIV) has not been realised, with virologic failure and progression to AIDS being detected in 7% and 25% of adults and children individuals living with HIV on treatment, respectively [2, 3].

To address poor treatment outcomes, the World Health Organization (WHO) recommended the use of Integrase Strand Transfer Inhibitors (INSTIs)-based regimen as a first-line treatment regimen for HIV in low-income countries [4]. In line with this recommendation, Tanzania introduced a dolutegravir-based fixed drug combination as a default first-line regimen in Tanzania’s HIV treatment program on the 1^st^ of March 2019. The dolutegravir-based regimens are known to rapidly suppress HIV viral load in treatment naïve and experienced patients [5–10]. Furthermore, evidence suggests that dolutegravir is well tolerated and demonstrates superior virological suppression compared to other ART classes in both treatment-naive and experienced patients, when it is used in combination with nucleoside reverse transcriptase inhibitors (NRTI) backbone [10–12]. In addition, dolutegravir has demonstrated a high genetic barrier to the development of HIV drug resistance [13, 14].

In Tanzania, the accelerated transition to the INSTI-based regimen involved initiation of a dolutegravir-based fixed combination regimen to all treatment naïve individuals as well as treatment-experienced eligible individuals on first-line ART regimen (NNRTI-based, PI-based or other ART combinations). The adopted dolutegravir transition policy in Tanzania does not require confirmation of virological suppression before switching to a dolutegravir-based regimen. This approach has been associated with dolutegravir treatment failure elsewhere as a result of the existing NRTI drug resistance mutations that subject clients to DTG monotherapy and virologic failure [15–18]. Additionally, HIV viral diversity, the client’s psychosocial factors and other host genotypic factors such as unique HLA class I alleles in the Tanzanian settings may influence the effectiveness of the dolutegravir-based regimen [19–21]. Therefore, 18 months after the introduction of the dolutegravir-based regimen we sought to conduct a nationally representative cross-sectional survey to determine the current HIV suppression rates in adults and children on ART in Tanzania. We report data on the viral suppression rates among adults and children PLHIV on ART and the factors associated with the lack of viral suppression in Tanzania. The current study findings provide information on the effectiveness of Tanzania’s ART program.

## Materials and methods

### Study design and settings

This survey used a cross-sectional study design as per the WHO HIV drug resistance surveillance protocol [22]. The protocol allows for the estimation of prevalence rates of HIV viral suppression at an early time point, 9–15 months and late time points on ART (≥ 36 months for children and ≥ 48 months for adults) [22]. The survey involved 36 healthcare facilities across 22 regions of Tanzania’s mainland. The survey was conducted from September to December 2020. The detailed sampling techniques used in this survey have been reported [23]. Briefly, a cluster sample design was employed in selecting survey sites from the list of 2,620 ART health facilities in Tanzania mainland that provide ART services to adults and children whose number per site was fixed. The sampling frame included 982 health facilities offering services to 90% of the HIV+ population who were on treatment during the data collection period. Probability proportional to size (PPS) was used in selecting the health facilities. A sampling interval of 21,359 was determined by dividing the cumulative number (768,907) of current PLHIV on ART and the number of facilities (36). The number 12,628 was used to select the first site after the sampling interval was entered into a random number generator. The total sample size of the participants for the cross-sectional survey was 2,160 and included all PLHIV on ART for a minimum of 9 months. This figure was purposefully inflated to ensure adequate enrolment of participants who belonged to the populations of interest, early time point, all persons enrolled in ART for 9–15 months and late time point comprising of adults enrolled on ART for a minimum of 48 months, and children enrolled on ART for a minimum of 36 months. Individuals who were on ART for less than 9 months were excluded from the survey.

### Survey population

We enrolled PLHIV in Tanzania mainland. The inclusion criteria used were adults aged 15 years and above and children aged 14 years and below attending HIV care and treatment centres (CTCs) at selected study sites. The detailed inclusion criteria used in this survey have been previously reported [23].

### Sample size estimation

The estimated sample size for the survey was 2,160 PLHIV obtained using the effective sample size formula: (N_eff_) = (t_df,0.975_)^2^ P_VL_ (1-P_VL_)/L^2^, where; where; t = degrees of freedom, P_VL_ = assumed prevalence of VS among Children and adolescents receiving ART for 9–15 months and L = half-length of confidence interval. The assumptions were that HIV viral suppression (VS) among adults was 87% while for children was 83.5%, according to the Tanzania HIV Impact survey 2016–2017 [24]. Given our sampling approach, we considered the design effect to get the appropriate sample size per site. The design effect (DEFF_info_) for probability proportion to the sampling approach was 1.5. The intra-cluster correlation for VL (ICC_VL_) for a site was 0.004 for the 9–15 and 36/48-month time points. Thus, the number of patients sampled per site was calculated using the formula1-ICC_VL_/(n/DEFF_info_*N_eff_)—ICC_VL._

### Data collection

Demographic data such as age, sex, marital status, education level, occupation and number of children were collected from participants using a questionnaire embedded through the Open Data Kit (ODK) platform in mobile Android-based tablets. Clinical information including ART regimens, and the previous and recent HIV viral load (HVL) test results were extracted from the electronic registers available at the surveyed sites (CTC2 database). Data on ART adherence was collected using a customized survey tool adapted from the Tanzania National guidelines for management of HIV and AIDS [25]. The tool was directly administered to the eligible adult participants (clients), and for the case of children, the tool was administered to a parent or guardian/caretaker. The adherence tool had a set of four questions of “Yes” and “No” responses that assessed self-reported ART adherence. These questions were set to enquire client’s negative experiences in taking ART medicine and the history of stopping taking medication. The response of “No” to all questions was defined as a high adherence, “Yes” to one question was defined as moderate adherence and “Yes” to two or more questions was defined as a low adherence. The scores were compared with adherence records available on the client’s CTC2 card.

In addition, 10 mL venous blood was collected into ethylenediamine tetraacetic acid (EDTA) vacutainer tubes (purple) using standard procedures. Samples were sent to the survey-designated laboratories for plasma preparation and temporary storage at either -20°C or -80°C freezer before shipment to Temeke Regional Referral Hospital Laboratory (TRRHL) for HVL testing [23].

### HIV viral load (HVL) testing

HVL was estimated from plasma samples following standard routine methods and logistics for HVL testing in the country. Viral load estimation in copies/mL was performed using the country-approved platform COBAS 8800 TaqMan (Roche Molecular system) available at the TRRHL [26]. The internal quality control was conducted according to the manufacturer’s instructions. The patterns of HIV viral suppression rates were classified as undetectable <50 copies/mL, suppression <1000 copies/mL, unsuppressed **≥**1000 copies/mL and lack of viral suppression to undetectable level **≥**50 copies/mL [27].

### Data management and analysis

Demographic information was merged with clinical data from the CTC2 database and exported into Microsoft Excel v.2016. Data was cleaned and analysed using Stata version 15.1 software and GraphPad Prism Software version 6.07 (GraphPad Software, San Diego, California, USA). Continuous variables were categorized, and descriptive statistics were used to characterize the study population. The estimates of overall viral suppression for the population of interest were calculated according to the participant’s characteristics. The chi-square test was used to examine statistical differences between categorical variables. Univariable and multivariable logistic regression models were used to determine the factors independently associated with the viral detectable levels (≥50 copies/mL). The factors with a p-value ≤ 0.05 were subjected to multivariable logistic regression analysis. A p-value of less than 0.05 was considered statistically significant for all statistical tests.

### Ethical consideration

The protocol was approved by the National Health Research Ethics Committee (NatHREC) of the National Institute for Medical Research (NIMR), Tanzania with reference number NIMR/HQ/R.8a/Vol.IX/3432. The permission to conduct the survey was also obtained from the President’s office- Regional Administration and Local Government (PORALG). A written informed consent was obtained from all survey participants aged 18 years and above and parents or guardians of participants aged below 18 years. In addition, a written assent was also obtained where applicable from participants aged 15 to 17 years.

## Results

### Demographic characteristics of the survey participants

A total of 2,039 PLHIV receiving care and treatment services were recruited. Of these, 1,173 were adults with a mean age and standard deviation (SD) of 42.1±12.4 years, and 64.7% were female. In contrast, children were 866, females contributed 53.2% and their mean age was 9.6±3 years. Most participants were in the late time point category of ART use where; 58.8% of adults had been on ART for at least 48 months, and over three-quarters (80.9%) of children were on ART for at least 36 months (Table 1). This survey was conducted eighteen months after the dolutegravir rollout in Tanzania. Among participants whose current ART regimen records were available (94.3% of adults and 92.3% of children) the majority (93.3%) of adults and 64.4% of children, were already transitioned to a dolutegravir-based regimen. Other demographic and clinical characteristics of the participants are summarised in Table 1.

**Table 1:** Demographic and clinical characteristics of survey participants (N = 2039).

Characteristic	Adults Frequency (%) N = 1173	Children Frequency (%) N = 866
**Age (years)**		** **
Mean age (SD)	42.1 (±12.4)	9.6 (±3)
1–7	*NA*	247 (28.5)
8–10	*NA*	221 (25.5)
10–14	*NA*	398 (46.0)
15–25	116 (9.9)	*NA*
26–30	83 (7.1)	*NA*
31–35	143 (12.2)	*NA*
36–45	376 (32.0)	*NA*
45+	455 (38.8)	*NA*
**Sex**		** **
Female	761 (64.9)	461 (53.2)
Male	412 (35.1)	405 (46.8)
**Level of education**		** **
None	287 (24.5)	450 (51.9)
Primary	735 (62.6)	393 (45.4)
Secondary	131 (11.2)	23 (2.7)
Post-secondary	20 (1.7)	*NA*
**Marital status**		
Single (never married)	183 (15.6)	*NA*
Married/cohabiting	659 (56.2)	*NA*
Divorced/Separated/widowed	331 (28.2)	*NA*
**Occupation (Means of earning income)**		
Unemployed	135 (11.5)	*NA*
Self-Employed	938 (80.0)	*NA*
Employed	100 (8.5)	*NA*
**Number of children**		* *
None	143 (12.2)	*NA*
1–2	390 (33.3)	*NA*
3–5	479 (40.8)	*NA*
6–9	146 (12.5)	*NA*
10+	15 (1.2)	*NA*
**Duration on ART**		** **
9–15 months	455 (38.8)	166 (19.2)
48+ months	718(61.2)	*NA*
36+ months	*NA*	700 (80.8)
**Current ART regimen**		
1^st^ line NNRTI-based	34 (3.1)	30 (3.8)
1^st^ line INSTI-based	1032 (93.3)	515 (64.4)
2^nd^ line PI-based	33 (3.0)	240 (30.0)
Other	7 (0.6)	14 (1.8)

NA-not applicable, DTG- Dolutegravir, NNRTI Non-Nucleoside reverse transcriptase inhibitor, PI- Protease inhibitor

### HIV viral load suppression rates among PLHIV on ART in Tanzania

**Fig 1:**
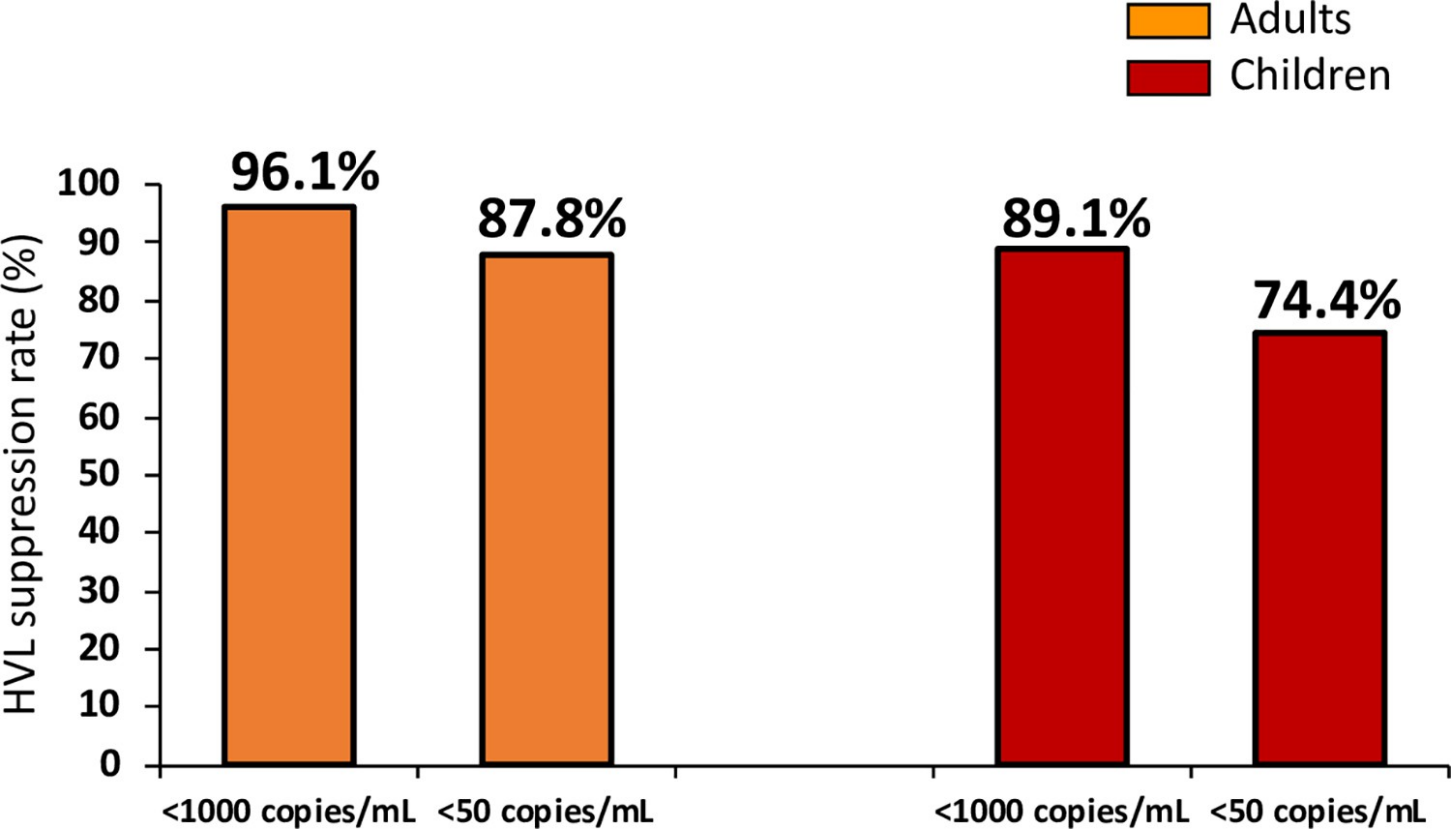
HIV viral suppression rates among adults and children in Tanzania. The bar graphs depict HIV viral suppression among the PLHIV adults and children on ART defined as HVL at a cut-off of 1000 copies/mL and <50 copies/mL as per the Tanzania National Guidelines for the Management of HIV and AIDS and the WHO recommended target.

Among 2005 participants with valid viral load results, 96.1% of adults and 89.1% of children had achieved a viral load suppression of <1000 copies/mL. However, only 87.8% of adults and 74.4% of children (Fig 1) had achieved the WHO-recommended and desired target for viral suppression of <50 copies/mL (undetectable).

### HIV viral load suppression patterns across participant’s demographic and clinical characteristics

In adult participants, we observed that the viral suppression rate at undetectable levels was higher in age groups above 26 years (88% to 90.5%) and the lowest (80%) in the age group of 15–25 years (p = 0.050). When we analysed the suppression rate according to current ART, the suppression rate by having undetectable levels (< 50 copies/mL) was higher (**≥**90%) among participants on first-line ART (NNRTI (94.1%) and DTG (89.7%)) based regimens. In contrast, those on second-line ART, PI-based and other regimens showed suppression rates to undetectable levels of <70% (p<0.0001). A high suppression rate to undetectable levels (90.4%) was observed among adults with high ART adherence while those ranked to have low ART adherence only 62.5% had viral suppression (p<0.0001) (Table 2).

**Table 2:** HIV viral load suppression among adults on ART in Tanzania.

Characteristics	Participants with viral load (copies/mL)		
	≥50 (Detectable) n (%)	< 50 (Undetectable) n (%)	_*X*_^*2*^ *p-value*
**Age (years)**			
15–25	23 (20.0)	92 (80.0)	**0.050**
26–30	9 (11.1)	72 (88.9)	
31–35	16 (11.2)	127 (88.8)	
36–45	35 (9.5)	335 (90.5)	
45+	54 (12.0)	395 (88.0)	
**Sex**			
Female	84 (11.2)	665 (88.8)	0.135
Male	59 (14.4)	350 (85.6)	
**Marital status**			
Single (never married)	27 (15.1)	152 (84.9)	0.189
Married/cohabiting	68 (10.4)	585 (89.6)	
Divorced/Separated/widowed	34 (10.4)	292 (89.6)	
**Occupation**			
Unemployed	13 (10.1)	116 (89.9)	0.883
Self-Employed	104(11.2)	821 (88.8)	
Employed	10 (10.1)	89 (89.9)	
**Duration on ART**			
9–15 months	55 (12.2)	394 (87.8)	0.115
48+ months	65 (9.17)	644 (90.8)	
**ART treatment status**			
Treatment naïve initiated to DTG	25 (13.3)	163 (86.7)	0.168
Treatment experienced switched to DTG	81(9.6)	763 (90.4)	
**Current ART regimen**			
1^st^ line NNRTI-based	2 (5.9)	32 (94.1)	**<0.0001**
1^st^ line DTG-based	106 (10.3)	926 (89.7)	
2^nd^ line PI-based	10 (30.3)	23 (69.7)	
Other	3 (42.9)	4 (57.1)	
**ART adherence**			
High	94 (9.6)	882 (90.4)	**<0.0001**
Moderate	19 (18.6)	83 (81.4)	
Low	30 (37.5)	50 (62.5)	

DTG- Dolutegravir, NNRTI- Non-Nucleoside reverse transcriptase inhibitor, PI- Protease inhibitor, and _X_^2^ indicates Chi-square.

Among children, the viral suppression rates to undetectable levels were higher among female children (77.3%), and those on ART for 36 months and above (76.2%) (p <0.05). Among those on first-line ART showed the highest suppression rates to undetectable levels (84.5%); those on dolutegravir had a suppression of 85.1% and NNRTI-based regimen 76.7%. In contrast, those on second-line PI-based and other regimens showed the lowest proportions of undetectable levels (<69.6%) (p<0.0001). Additionally, children with high adherence had higher (85.1%) viral suppression to undetectable levels while for those ranked as having moderate and low adherence the suppression rates were 31.0% and 13.1% respectively (p<0.0001) (Table 3).

**Table 3:** HIV viral load suppression among children on ART in Tanzania.

**Characteristic**	**Participants with viral load (copies/mL)**		
	**≥50 (Detectable)** **n (%)**	**< 50 (Undetectable)** **n (%)**	_***X***_^***2***^ ***p-value***
**Age (years)**			
1–7	68 (28.5)	171 (71.5)	0.403
8–10	56 (26.0)	159 (74.0)	
11–14	93 (23.7)	300 (76.3)	
**Sex**			
Female	103 (22.7)	350 (77.3)	**0.047**
Male	114 (28.9)	280 (71.1)	
**Duration on ART**			
9–15 months	54 (33.3)	108 (66.7)	**0.016**
36+ months	163 (23.8)	522 (76.2)	
**ART treatment status**			
Treatment naïve initiated to DTG	10 (34.5)	19 (65.5)	**0.006**
Treatment experienced switched to DTG	74 (13.9)	460 (86.1)	
**Current ART regimen**			
1^st^ line NNRTI-based	7 (23.3)	23 (76.7)	**<0.0001**
1^st^ line DTG-based	84 (14.9)	479 (85.1)	
2^nd^ line PI-based	73 (30.4)	167 (69.6)	
Other	5 (35.7)	9 (64.3)	
**ART adherence**			
High	111 (14.9)	633 (85.1)	**<0.0001**
Moderate	29 (69)	13 (31.0)	
Low	53 (86.9)	8 (13.1)	

DTG- Dolutegravir, NNRTI- Non-Nucleoside reverse transcriptase inhibitor, PI- Protease inhibitor and _X_^2^ indicates Chi-square.

### Determinants for lack of viral suppression to undetectable levels among PLHIV in Tanzania

All the variables that had a p-value of ≤ 0.05 in univariable analysis were subjected to multivariable analysis. On multivariable analysis age and ART adherence were the factors independently associated with having a detectable viral load of **≥** 50 copies/mL status among adults living with HIV. Adults aged 15–25 years had 2.41 higher odds of lack of viral suppression compared to those aged 45 years and above (aOR = 2.41, 95% CI = 1.34–4.32). Adults with moderate and low ART adherence had significantly higher odds of lack of viral suppression compared to those with high ART adherence aOR = 3.01, 95% CI = 1.77–5.13 and aOR = 8.05 95% CI = 4.75–13.65, respectively (Table 4).

**Table 4:** Factors associated with lack of viral suppression among adults living with HIV in Tanzania.

Variable	Bivariate analysis		Multivariate analysis	
	cOR (95% CI)	p-value	aOR (95% CI)	p-value
**Age (years)**				
15–25	2.72 (1.61–4.58)	<0.001	2.41 (1.34–4.32)	**0.003**
26–30	0.90 (0.41–1.98)	0.796	0.95 (0.42–2.17)	0.907
31–35	0.22 (0.68–2.17)	0.508	1.24 (0.68–2.29)	0.483
36–45	0.90 (0.57–1.43)	0.660	0.87 (0.54–1.41)	0.577
>45	Ref		Ref	
**Adherence status**				
High	Ref		Ref	
Moderate	2.94 (1.74–4.95)	<0.001	3.01 (1.77–5.13)	<**0.001**
Low	8.24 (4.94–13.75)	<0.001	8.05 (4.75–13.65)	<**0.001**
**Current ART regimen**				
1^st^ line DTG-based	0.37 (0.16–0.86)	0.020	0.45 (0.18–1.12)	0.086
1^st^ line NNRTI-based	Ref		Ref	
2^nd^ line PI-based	0.26 (0.06–1.09)	0.066	0.27 (0.06–1.22)	0.089
Other	0.48 (0.05–4.61)	0.524	0.73 (0.07–7.40)	0.791

DTG-Dolutegravir, NNRTI- Non-Nucleoside reverse transcriptase inhibitor, PI- Protease inhibitor, HVL- HIV viral load, OR-Crude Odds Ratio, aOR- Adjusted Odds Ratio, CI- Confidence Interval

Among children, male children had higher odds (aOR = 1.41, 95% CI = 1.02–1.95) of lack of viral suppression having a detectable viral load of **≥** 50 copies/mL compared to female children. Children on 1^st^ line NNRTI-based and 2^nd^ line PI-based regimens had higher odds (aOR = 2.58, 95% CI = 1.19–5.61) and (aOR = 1.67, 95% CI = 1.18–2.38), respectively of lack of viral suppression compared to those on 1^st^ line DTG-based regimen. Additionally, low ART adherence in children was also associated with increased odds of lack of viral suppression to undetectable levels compared to those with high ART adherence (aOR = 2.21 95% CI = 1.26–3.89) (Table 5).

**Table 5:** Factors associated with lack of viral suppression among children living with HIV in Tanzania.

Variable	Bivariate analysis		Multivariate analysis	
	cOR (95% CI)	p-value	aOR (95% CI)	p-value
**Sex**				
Male	1.38 (1.00–1.90)	0.049	1.41 (1.02–1.95)	**0.038**
Female	Ref		Ref	
**Duration on ART**				
9–15 months	1.32 (0.89–1.95)	0.162	1.40 (0.94–2.09)	0.096
>36 months	Ref		Ref	
**Adherence status**				
High	Ref		Ref	
Moderate	1.58 (0.76–3.29)	0.217	1.58 (0.75–3.31)	0.230
Low	2.03 (1.17–3.52)	0.012	2.21 (1.26–3.89)	**0.006**
**Current ART regimen**				
1^st^ line DTG-based	Ref		Ref	
1^st^ line NNRTI-based	2.33 (1.08–5.04)	0.031	2.58 (1.19–5.61)	**0.017**
2^nd^ line PI-based	1.66 (1.17–2.35)	0.004	1.67 (1.18–2.38)	**0.004**
Other	1.10 (0.30–4.00)	0.887	0.84 (0.23–3.16)	0.801

DTG-Dolutegravir, NNRTI- Non-Nucleoside reverse transcriptase inhibitor, PI- Protease inhibitor, HVL- HIV viral load, OR-Crude Odds Ratio, aOR- Adjusted Odds Ratio, CI- Confidence Interval

## Discussion

Tanzania is among the top 15 countries with the highest burden of HIV infection in the world. Since the national ART program was initiated in 2007, considerable progress has been made towards achieving the 95-95-95 UNAIDS goals for diagnosis, linkage to HIV treatment, and viral suppression rates in PLHIV. However, the previous 2016–2017 national HIV impact survey indicated that Tanzania had yet to achieve the first and third goals for both children and adults living with HIV [24].

In this study, we present the first results of the healthcare facility-based HIV viral suppression survey conducted since the 2016–2017 survey [24]. This nationally representative survey took place 18 months after the introduction of dolutegravir in Tanzania. Our findings indicate that 96.1% of adults and 89.1% of children had achieved a viral load suppression of <1000 copies/mL. These findings indicate that Tanzania has yet to attain the UNAIDS goal of ensuring at least 95% of PLHIV for the children population. The present findings highlight the need to closely follow and properly manage children on ART to suppress the viral load in our setting. Furthermore, our analysis shows that HIV viral suppression rates defined at <50 copies/mL (undetectable) as recommended by WHO were 87.8% in adults and 74.4% in children on treatment. These results indicate that even after a year and a half of dolutegravir introduction in the country, the desired target of viral suppression (<50 copies/mL) has not yet been achieved. These findings suggest the existence of some barriers such as ART adherence in the progress towards optimum treatment outcome in PLHIV who have transitioned to the dolutegravir-based regimen.

The rapid transition to a dolutegravir-based fixed combination as the default first-line regimen in Tanzania is expected to improve virological suppression rates [28–30]. In this survey, 93.3% and 64.4% of the recruited adults and children, respectively, had already transitioned or were initiated to the dolutegravir-based regimen and their respective viral suppression rates stood at 89.7% for adults and 85.1% for children. This finding is similar to what has been reported in Malawi whereby suppression of children on dolutegravir was 81.5% [31]. Previous studies have shown that dolutegravir-based regimens have better viral suppression rates in treatment-naïve compared to treatment-experienced PLHIV [7, 32]. However, in our study, we did not observe a statistically significant difference in adults initiated to the dolutegravir-based regimen compared to those switched to the dolutegravir-based regimen. In contrast, children initiated with the dolutegravir-based regimen showed a worse suppression rate compared to those switched to the dolutegravir regimen. These observations have also been reported in other studies where treatment-naive showed relatively lower viral suppression rates compared to treatment experienced after using a dolutegravir fixed combination [33, 34], interestingly in one study, self-reported treatment naïve individuals who had baseline <50 copies/mL ended up with high viremia following initiation with dolutegravir treatment [33]. These data suggest that in real-world scenarios the treatment naïve, silent-client transfers (with ART exposure and HIV drug resistance) and treatment experience groups may have differences in characteristics including treatment adherence which could influence treatment outcomes on a dolutegravir-based regimen [5, 35]. Understanding the drivers for silent-client transfer and outcomes in viral suppression may help ART programs understand and prioritize opportunities for improvement. Furthermore, HIV viral diversity and subtype may impair the effectiveness of the dolutegravir-based regimen; nevertheless, other client factors such as suboptimal ART dose, family support, gender, marital status, and level of education may also interfere with ART adherence and hence may affect the effectiveness of the dolutegravir-based regimen [20, 21, 31, 36, 37]. In addition, emerging evidence on host genotypes such as HLA class I alleles suggests the existence of unique HLA-I alleles (A*66:01, A*68:01, A*68:02, A*30:04, B*15:17, B*35:02, B*15:03, and C*17:01) in the African population that are positively correlated with INSTI resistance (T97A, E138K and R263K) [19]. These factors along with the adopted dolutegravir transition strategy and policy in Tanzania which does not require confirmation of virological suppression before switching to a dolutegravir-based regimen may contribute to viral suppression trends observed in our survey. The transitioning strategy has been associated with dolutegravir treatment failure due to DTG monotherapy as a result of pre-existing NRTI drug resistance mutations [15–18]. This trend of virologic failure among PLHIV on dolutegravir-based regimens has been reported in similar settings such as the Malawian population [18]. Additional studies are required to understand the dynamics of dolutegravir effectiveness in different settings especially in children initiated to this regimen.

Compared to NNRTI and INSTI-based regimens, the rates of viral suppression were the lowest in both children and adults receiving PI-based regimen. This may be due, at least in part, to the fact that PI-based regimens are often used as a second-line treatment for individuals who have failed first-line regimens [38], and pre-existing individual factors associated with poor treatment outcome may persist during treatment with a second-line line PI-based regimen. However, further research is needed to understand the underlying factors behind this observation and to identify strategies for improving treatment outcomes among individuals receiving PI-based regimens.

Several independent risk factors for lack of suppression were identified in this study. We found that both adults and children, having moderate to low ART adherence have higher odds of lack of viral suppression to undetectable levels compared to those with high ART adherence. These findings cement the role of high ART adherence in achieving HIV viral suppression among PLHIV [36, 37, 39, 40]. Moreover, our findings concur with previous studies that have reported sub-optimal adherence is associated with a lack of viral suppression among PLHIV on a DTG based in Tanzania and other settings [37, 41, 42]. We also observed that adults aged 15–25 years had higher odds of lack of suppression compared to those aged 45 years and above. Our finding is similar to other studies which found that adolescents and younger adults tend to have lower suppression rates compared to the older age groups [33]. These findings are similar to those reported in Tanzania and other settings [42, 43]. Adolescents and young adults face several issues including psychosocial ones that may lead to the struggle to remain engaged in HIV care and treatment which subsequently may affect ART adherence [20, 44, 45]. Major psychological and social factors such as stigma, social support, substance abuse, subjective norms, belief systems and perceived behavioural control are some of the potential barriers to ART including dolutegravir adherence [46–48].

We also observe that similar to adult populations, male children had higher odds of lack of viral suppression to undetectable levels compared to females, this trend is similar to previous studies conducted in similar settings [20, 44, 45, 49–51]. These findings suggest similar drivers for non-suppression among adult males which include poor ART adherence and poor treatment-seeking behaviours may also contribute to older male children. This trend concurs with previous studies that have shown dolutegravir effectiveness in viral suppression in this group of people living with HIV [6, 45].

Further studies to determine the contribution of psychosocial and cultural factors on dolutegravir effectiveness in viral suppression are warranted.

Evidence presented in this survey should be discussed in light of study limitations. First, the viral load (VL) analysis was based on a single HIV viral test taken during the study period, which may not accurately reflect virologic failure but rather high viraemia. However, the quality control measures in place give us confidence in this single measure for high viraemia. In addition, this was a cross-sectional study and hence it was not possible to effectively directly assess the effectiveness of dolutegravir-based regimen on viral suppression. In particular, HVL data at the time of switching to a dolutegravir-based regimen were not available for most participants to directly analyse the effectiveness of dolutegravir. Moreover, the duration post-switching to dolutegravir varied widely between participants. Furthermore, due to study duration limitation, the target sample of 2,160 was not reached; instead 2039 (94.4%) participants were recruited. Despite these limitations, this study has significant strengths, our survey is the first and the largest, nationally representative survey that included both children and adult populations of PLHIV in Tanzania hence eliminating potential bias in participant selection. Our data provide insights into the state of viral suppression as per the desired WHO target of undetectable viral load in Tanzania. We also show the important program factors such as ART adherence that should be strengthened to ensure the success of dolutegravir-based regimen transition in Tanzania.

## Conclusion

The present study highlights that the country still faces challenges in achieving the third 95 of the 95-95-95 UNAIDS goal. Our findings suggest that the transition to dolutegravir-based regimens has the potential to improve viral suppression rates in PLHIV. However, there are complexities in the effectiveness of this regimen, between groups of PLHIV. The differences observed, particularly in children, emphasize the need for further research to understand the factors influencing treatment outcomes in diverse settings. Efforts to attain epidemic control by ensuring VL suppression should focus on the identified factors that are associated with lower rates of viral suppression.
